# Cognitive, language and motor developmental patterns of extremely preterm children up to 2 years of age: a descriptive approach

**DOI:** 10.3389/fpsyg.2025.1599390

**Published:** 2025-07-02

**Authors:** Erika Jurišová, Lucia Ráczová, Marta Zaťková, Martina Romanová

**Affiliations:** ^1^Department of Psychological Sciences, Faculty of Social Sciences and Health Care, Constantine the Philosopher University in Nitra, Nitra, Slovakia; ^2^Institute of Applied Psychology, Faculty of Social Sciences and Health Care, Constantine the Philosopher University in Nitra, Nitra, Slovakia

**Keywords:** extremely preterm children, cognitive development, language development, motor development, developmental patterns

## Abstract

**Background:**

Advancements in technology and pharmacology over the past 15 years have increased the survival rates of extremely low gestational age newborns to over 80%. However, these medical achievements are often accompanied by significant challenges in their early and long-term developmental competencies. This longitudinal descriptive study aimed to examine the neurodevelopmental patterns, prevalence of developmental delays, and associated risk factors –gestational age (GA) and birth weight (BW)—in Slovak children born at extremely low gestational age (ELGA), from 7 to 8 months of corrected age to 24–25 months of chronological age, focusing on cognitive, motor, and language development.

**Methods:**

The study included 7 female and 10 male ELGA children with a mean GA of 26.0 weeks (*SD* = 1.2; range = 24–28) and mean BW of 875.8 grams (*SD* = 171.2; range = 560–1,150). The Bayley Scales of Infant and Toddler Development, Third Edition (Bayley-III) was administered to assess cognitive, language, and motor development. The developmental functioning of cognitive, linguistic, and motor skills was analyzed using a descriptive approach, based on the average composite scores attained in each domain, in comparison with the normative group defined by the Bayley-III. The developmental patterns of cognitive, linguistic, and motor skills in the observed ELGA children were constructed based on the level of composite scores at two time points: the 7th or 8th month of corrected age (initial assessment) and the 24th or 25th month of chronological age (final assessment). These performance values were classified according to the ‘cut-off’ criteria for developmental delay in the Bayley-III. The degree of delay at these two time points determined the type of developmental pattern.

**Results:**

The developmental functioning of cognitive, language, and motor skills in the sample of ELGA children studied at the 7th or 8th month of corrected age was within the average range. However, the average scores of ELGA children were 5 to 10 points lower than those of the normative population across domains. At this time point, only 30% of the ELGA children exhibited developmental delay in at least one domain, exclusively at the level of mild to moderate delay (< −1 SD). The prevalence of mild to moderate delay (< −1 SD) was as follows: cognition: 11.7%, language: 11.7%, motor: 29.4%. In the studied sample, we observed a decline in cognitive, language, and motor functioning to the low-average range at 2 years of chronological age. The average scores of ELGA children were 12 to 17 points lower than those of the normative population across domains. At this point, up to 58.9% of the ELGA children exhibited developmental delay in at least one domain. The prevalence of developmental delay was as follows: mild to moderate delay (< −1 SD) was observed in 29.4% of children for cognition, 29.4% for language, and 17.6% for motor skills. Severe delay (< −2 SD) was present in 11.7% of children for cognition, 17.6% for language, and 17.6% for motor skills. In the sample of children with ELGA, we observed declining developmental trends in cognitive and motor skills; however, the values of developmental functioning remained within the range of typical development without developmental delay. Regarding language skills, we observed the most pronounced decline during the first 2 years of development, shifting from typical development to a mild-to-moderate delay (< −1 SD). Within individual domains, we identified four types of developmental patterns in cognitive, language, and motor skills among ELGA children. These patterns were as follows: (1) ascending into the normal range: 0, 5.8, and 11.7%, respectively; (2) stable pattern within the normal range: 58.8, 47.0, and 52.9%; (3) stable pattern within the delayed range: 11.7, 5.8, and 17.6%; (4) descending into the delayed range: 29.4, 41.1, and 17.6%. Findings indicate a higher incidence of developmentally risky patterns in children born at low gestational age (24–25 weeks) and in children with birth weight below 750 grams and lower. Developmental functioning without delay in all three domains (cognitive, language, and motor) was observed in 41.1% of the ELGA children during their second year. The limitation of the study was the small sample size and the absence of Slovak standards for Bayley-III.

**Conclusion:**

The results demonstrate significant the need for early and long-term monitoring of developmental trends in this high-risk population, as well as the need to identify a broader range of health and non-health risk factors and their interactions that contribute to their final developmental outcomes.

## 1 Introduction

The preterm birth rate, as defined by the World Health Organization (WHO), refers to births occurring before 37 weeks of gestational age (GA) and is commonly used to classify different types of preterm children. Late (34–36 weeks, 60%) and moderate preterm births (32–33 weeks, 20%) are more common, whereas very preterm (28–31 weeks, 15%) and extremely preterm (< 28 weeks, 5%) account for about one in five preterm births (Goldenberg et al., 2008). The incidence of preterm births and survival rates of *extremely low gestational age newborns* (ELGA) have increased to over 80% in the past 15 years due to advances in obstetrics and neonatal intensive care (Sansavini et al., 2014). The latest data on the survival rate of ELGA children in Slovakia dates back to 2021, when 13.3% of births were preterm, with 0.34% of them being live-born ELGA infants.

### 1.1 Cognitive, language and motor development among preterm children in short-and long-term outcomes

Preterm birth interrupts the natural course of fetal development and forces the immature brain and body to adapt to an artificial environment the neonatal intensive care unit (NICU). In this setting, newborns are exposed to both overstimulation (e.g., bright lights, loud noises, and pain from invasive medical procedures) and understimulation (due to the lack of prenatal rhythmic and kinesthetic input, continuous contact with the mother, and restricted movement caused by incubator positioning and medical care) (Sansavini et al., 2011). Recent research has revealed the negative consequences of early visual deprivation in terms of weakened face detection in preterm infants undergoing phototherapy due to hyperbilirubinemia (de Almeida et al., 2025). Preterm birth is associated with neurological damage (e.g., cerebral palsy, periventricular leukomalacia, intraventricular hemorrhage, hypoxic–ischemic encephalopathy, hydrocephalus) (Sansavini et al., 2011) and neurosensory disabilities (e.g., blindness, deafness) (Aylward, 2009). Proximal (related to relationships) and distal (related to socioeconomic status) social factors have also been described as influencing the development of preterm infants (Aylward, 2009).

The reduction in mortality has resulted in a higher incidence of short-and long-term morbidities, as well as neurodevelopmental sequelae, impacting the developmental functioning of survivors (Sansavini et al., 2014). Early consequences of prematurity include developmental difficulties, deviations in developmental functioning and psychomotor development, as well as neurodevelopmental sequelae and disorders that manifest before 36 months of age. Compared to more mature neonates, ELGA children have a higher incidence of neonatal morbidity (Blakely et al., 2005; Laptook et al., 2005; Shankaran et al., 2004; Vohr et al., 2004; Walsh et al., 2005), a higher prevalence of complex chronic conditions (respiratory, gastrointestinal, and growth delays), and an increased risk of long-term and persistent mild neurobehavioral and cognitive deficits (neurological, sensory, cognitive, and behavioral difficulties) (Laptook et al., 2005; Shankaran et al., 2004; Vohr et al., 2003; Walsh et al., 2005; Vohr et al., 2005; Costeloe et al., 2012; Johnson and Marlow, 2017). ELGA children exhibit high levels of dysfunction in various cognitive domains, such as attention, visual processing, and executive functioning (Anderson et al., 2004; Jaekel et al., 2013; Vicari et al., 2004). Gardon et al. (2019) found that children with extremely low birth weight (ELBW) at 2 years of corrected age exhibited delays in expressive communication, with a ratio of 25.3% (< −1 SD) and 11.1% (< −2 SD). Other studies also point to delays in language skills in ELGA children at 2 years of age (Foster-Cohen et al., 2007; Gayraud and Kern, 2007), especially in boys aged 2.5 y ears in expressive communication (Sansavini et al., 2006). Pierrat et al. (2017) reported that preterm children are at a higher risk of delays in gross motor skills and language development. At 2 years of corrected age, delays in gross motor skills were observed in 16.6% of children born at 24–26 weeks of GA, 9.7% of children born at 27–31 weeks of GA, and 5.1% of children born at 32–34 weeks of GA. At 2 years of corrected age, delays in expressive communication were observed in 33.9% of children born at 24–26 weeks of GA, 24.1% of children born at 27–31 weeks of GA, and 17.8% of children born at 32–34 weeks of GA. Due to prematurity or low birth weight, language development may be weakened, manifesting as expressive and/or receptive difficulties (Selassie et al., 2005). Difficulties can be observed in verbal fluency, sound imitation, and auditory discrimination (Jennische and Sedin, 2001). These difficulties are likely a consequence of a global deficit rather than a specific disorder (Wolke et al., 2008).

Follow-up studies of children born at ELGA have documented a broad spectrum of neurodevelopmental difficulties. These include challenges with self-regulation and increased incidence of hyperactive or aggressive behaviors (Scott et al., 2012; Månsson and Stjernqvist, 2014), a higher prevalence of autism spectrum disorders (Stephens et al., 2012; Luyster et al., 2011), and early childhood difficulties in social interaction, attention, sleep, feeding, and sensory sensitivity (Wood et al., 2000; de Waal et al., 2012; Arpi and Ferrari, 2013). Additionally, ELGA children exhibit elevated rates of socio-emotional and adaptive functioning difficulties (Aarnoudse-Moens et al., 2009; Taylor et al., 2011; Lobo and Galloway, 2013; Peralta-Carcelen et al., 2017). Alterations in sensory development have also been observed in this population, particularly in sensory input, stimulation, and the regulation of sensory experiences (Rogers and Hintz, 2016). Because sensory processing forms a critical foundation for early learning, atypical sensory sensitivities can have far-reaching consequences for brain development. Recent research has emphasized the significance of face perception and processing in early infancy as a potential early marker of neurodevelopmental disorders (Simion and Giorgio, 2015). For example, studies have identified differences in visual social attention networks between newborns at high versus low risk for autism (Di Giorgio et al., 2016; Di Giorgio et al., 2021).

Children born at ELGA have also been found to be at increased risk for functional deficits during the school years, often requiring additional educational support. These difficulties tend to be subtle but include impairments in motor coordination, social-pragmatic communication skills, and cognitive performance particularly in working memory, problem-solving, and executive functioning. The prevalence of these deficits increases with decreasing gestational age and has been reported in up to 40% of children born before 26 weeks of gestation (Taylor et al., 2011; Msall, 2011; Squarza et al., 2017). Extreme prematurity has also been linked to significant psychosocial and emotional consequences for families. Higher levels of parental distress have been associated with lower household income, lower parental education, and greater severity of the child’s functional impairments (Singer et al., 1999; Cronin et al., 1995; Taylor et al., 2001).

Ongoing research is essential to generate up-to-date evidence on the short-and long-term neurodevelopmental outcomes of extreme prematurity, which is critical for developing clinical guidelines and delivering informed, evidence-based counselling to families.

### 1.2 Developmental trajectories and patterns among preterm children

Karmiloff-Smith (1998) states that understanding the consequences of preterm birth requires studying how children grow and change over time. Similarly, Thomas et al. (2009) emphasize that the most optimal way to understand developmental disorders is to design trajectory-based studies that assess how phenotypes gradually emerge over time and transform with age. Moreover, it appears that the developmental pathways of preterm children are atypical, not merely delayed, and are characterized by distinct developmental patterns and relationships between competencies (Sansavini et al., 2011).

Preterm children often experience delays in psychomotor development due to neonatal immaturity. A common phenomenon is the so-called cascading effect, which negatively impacts subsequent development. If a child fails to sufficiently develop certain cognitive, language, motor, or sensory abilities in the early stages of development, they may struggle to achieve subsequent milestones, leading to a worsening of the deficit. It is assumed that elementary cognitive functions influence more complex ones (Rose et al., 2011; Sansavini et al., 2011). Persistent psychomotor developmental delays often serve as precursors to neurodevelopmental disorders in later childhood.

Sansavini et al. (2014) identified the so-called Matthew Effect, which describes an increasing divergence in performance over time between preterm children and those born at term. By modeling growth curves using raw scores across different domains of the Bayley-III, they found that although the developmental trend was upward in all three domains (cognition, language, and motor skills), ELGA children consistently performed significantly lower and did not close this gap by 36 months. Similarly, Matthew Effects have been observed in other at-risk groups (e.g., children with language disorders) in later growth trajectories (Morgan et al., 2011).

Conversely, Lemola (2015) describes the opposite trend, arguing that some preterm children can compensate for their deficits in cognitive, language, and motor skills at a later age, a phenomenon referred to as the catch-up effect. This means that these children can achieve age-appropriate developmental outcomes. Thecatch-up effect in various developmental domains among preterm children has been confirmed in multiple studies (inhibition and cognitive flexibility: Everts et al., 2019; receptive communication: Luu et al., 2009; language skills: Nguyen et al., 2018). However, it is important to note that this effect has been observed predominantly at later ages (most often during school years). A possible explanation is brain plasticity, which may facilitate favorable neurocognitive development in preterm children, as well as the extended time required for the maturation of these functions, often persisting into early adulthood.

Similarly, Logan and Petscher (2010) describe four possible outcomes when examining early developmental trajectories in cognition, language, and motor skills among ELGA children compared to their full-term peers. Firstly, ELGA and full-term children may show no difference in initial status (intercept) or growth over time. Secondly, ELGA and full-term children may differ in their initial status but not in their growth rate, indicating that any differences observed at the final time point reflect the difficulties present at the beginning. Thirdly, ELGA and full-term children may have different growth rates, with two possible scenarios. One possibility is that ELGA children develop more rapidly than their full-term peers, following a compensatory developmental trajectory (Parrila et al., 2005). The other possibility is that ELGA children grow more slowly than full-term peers, demonstrating a Matthew Effect, in which they fall progressively further behind in early development. Fourthly, ELGA and full-term children may differ in both their initial status and their rate of growth over time. Despite the significant risk of complications in the perinatal period for preterm children, particularly for the high-risk ELGA group, predicting how these complications manifest in clinical variability and developmental outcomes remains challenging. Sansavini et al. (2011) emphasize that the developmental outcomes of preterm children are highly heterogeneous due to the complex interaction of biological and environmental constraints unique to preterm infants, as well as the timing of these influences.

Although gestational age is often a key determinant of survival and complications in preterm infants, more detailed prognostic assessments are increasingly focused on ELGA infants and/or newborns with a birth weight below 1,500 g. *Very low birth weight infants* (VLBW; < 1,500 g) and *extremely low birth weight infants* (ELBW; < 1,000 g) are at particularly high risk due to increased perinatal, neonatal, and postnatal mortality and morbidity (Korbeľ et al., 2014). The literature also describes further stratification of ELBW categories, including *very extremely low birth weight infants* (VELBW; < 750 g) and *fetal infants* (< 600 g) (Chovancová, 2025). Studies (Salas et al., 2016; UCSF Benioff Children’s Hospitals, n.d.) indicate that gestational age and birth weight are strong predictors of neurodevelopmental impairment (e.g., cognitive delays, cerebral palsy, and visual/auditory deficits) and mortality in preterm populations.

### 1.3 Objectives of this study

This longitudinal descriptive study aimed to examine the neurodevelopmental patterns, prevalence of developmental delays, and associated risk factors—specifically gestational age and birth weight—in Slovak children born at extremely low gestational age, from 7 to 8 months of corrected age to 24–25 months of chronological age, focusing on cognitive, motor, and language development.

We acknowledge that early developmental outcomes provide only a preliminary glimpse into the potential lifelong consequences of prematurity, which may persist into school age, adolescence, and even adulthood. Prematurity is recognized as an independent risk factor for adverse developmental outcomes; however, significant variability exists within this population regarding the types and severity of delays and impairments. Research suggests that early outcomes assessed between 18 and 36 months are not static and may not fully capture a child’s skills. For instance, cognitive skills have been shown to continue developing throughout childhood (Spittle et al., 2015; Vohr et al., 2003).

Conversely, several studies highlight the value of early developmental testing up to 36 months of age. For instance, the Bavarian Longitudinal Study demonstrated that cognitive skills assessments at 20 months of age in 260 very low gestational age children were significant predictors of IQ at 26 years of age (Breeman et al., 2015). Similarly, findings from the EPICure study (Marlow et al., 2005) indicate that, among ELGA children, BSID-II Mental Development Index (MDI) scores at −3 SD in early childhood strongly predict moderate to severe cognitive impairments by the age of 6.

In designing the research, we drew upon critical insights from Jary et al. (2011), which indicate that, unlike neurological examinations performed during the first months after birth, standardized developmental assessments using the BSID-II are highly reliable when conducted at 2 years of age in preterm infants. These assessments are particularly effective in detecting significant functional impairments in cognitive, language, and motor domains. The conclusion underscores the importance of early developmental assessment in infancy.

According to Johnson and Marlow (2006), the period following the second year of life is considered ideal for identifying neurodevelopmental delays in preterm children, as many conditions linked to preterm birth may not yet be evident earlier. Early identification of neurodevelopmental delays is critical, as it enables determination of the need for early intervention in this high-risk group of neonates. Similarly, several authors (Wolke et al., 2019; Kilbride et al., 2022; Garfinkle et al., 2024) consider this time point significant due to the increased likelihood of identifying developmental challenges and the potential for implementing targeted and early stimulation.

## 2 Method

### 2.1 Sample

The study included 17 Slovak ELGA children (7 female, 10 male) with a mean gestational age of 26.0 weeks (SD = 1.2; range = 24–28) and a mean birth weight of 875.8 grams (SD = 171.2; range = 560–1,150). Inclusion criteria were: (a) GA ≤ 28 weeks, determined by the date of the mother’s last menstrual period and confirmed by first-trimester early ultrasonography; (b) absence of major cerebral damage [e.g., periventricular leukomalacia (PVL), IVH grade > III, hydrocephalus] or congenital malformations; (c) no severe visual impairments [e.g., retinopathy of prematurity (ROP) grade > III] or hearing impairments. Only children whose primary home language was Slovak were included in the study, as research suggests that bilingualism is associated with slower cognitive and communicative-linguistic development in preterm children during the first 2 years of life (Walch et al., 2009; Sansavini et al., 2014).

Although children with severe cerebral damage or malformations were excluded from the study, certain health complications were still observed among the included participants. These complications included intraventricular hemorrhage (IVH) grade I–II (*n* = 5; 29%), retinopathy of prematurity (ROP) grade I–II (*n* = 7; 41%), bronchopulmonary dysplasia (BPD, *n* = 7; 41%), and sepsis (*n* = 8; 47%). Six children (35%) were from multiple pregnancies (twin births), and one child (6%) was conceived via *in vitro* fertilization and embryo transfer (IVF + ET). Mechanical ventilation (oxygen therapy) was required for 58.8% of the children, with a duration ranging from 4 to 60 days (mean duration = 17.9 days). Four children (23%) were delivered spontaneously in cephalic presentation, while 13 children (76%) were delivered via cesarean section. The participants were born between 2012 and 2020 and received care in three perinatology centers in Slovakia.

This study is a case series focusing on the developmental patterns of children born extremely preterm. The sample size (*n* = 17) includes all available cases with complete data on the development of their cognition, language and motor skills up to the second year of life. Because extremely preterm births are rare, the sample reflects the limited number of eligible participants rather than a predetermined size based on power analysis. Inferential statistical analyses were not performed due to the small sample. However, the study provides important descriptive insights into the development of this high-risk group.

### 2.2 Materials

*The Bayley Scales of Infant and Toddler Development, Third Edition* (Bayley-III, Bayley, 2006) was administered to assess cognitive, language, and motor development through three individual developmental scores: a cognitive composite score, a language composite score (with receptive and expressive subscores), and a motor composite score (with gross and fine motor subscores). Composite scores are derived from various sums of subtest scaled scores. The Bayley-III has been demonstrated to be a valid tool in both research and clinical practice; satisfactory reliability and validity are reported by the authors (Bayley, 2006), with test–retest reliability ranging from 0.6 to 0.9, internal consistency coefficients (using the split-half method) of 0.8–0.9, and moderate to high correlations with measures of similar domains. The Bayley-III has not yet been standardized in Slovakia. Therefore, in this study, we relied on the normative data published by the test authors (Bayley, 2006).

### 2.3 Procedure and data analysis

The aim of the study was to understand the developmental patterns of cognitive, language, and motor skills in ELGA children up to 2 years of age.

#### 2.3.1 Descriptive statistics and developmental level assessment

In the first step, we assessed the developmental level of cognitive, language, and motor skills at two key time points: at 7 or 8 months of corrected age and at 24 or 25 months of chronological age. Descriptive statistics were used to summarize the composite scores, including the mean, standard deviation (SD), median, and 95% confidence intervals (CI). To evaluate developmental functioning in the ELGA sample, we analyzed the average composite scores in each domain (cognitive, language, and motor) at both time points and compared them to the normative data provided by the Bayley-III (Bayley, 2006). The Bayley-III composite scores are standardized, with a mean of 100 and a standard deviation of 15, and range from 40 to 160. Children’s performance was classified according to established Bayley-III categories: very superior (≥ 130), superior (120–129), high average (110–119), average (90–109), low average (80–89), borderline (70–79), and extremely low (≤ 69) (Bayley, 2006). These classifications were used in line with recognized cut-off points for identifying developmental delays (Vohr et al., 2012). We also identified the prevalence of developmental delay at both time points based on the Bayley-III cut-off criteria (Vohr et al., 2012). A composite score below 70 (more than 2 SDs below the mean) indicates a significant developmental delay, while a score below 85 (more than 1 SD below the mean) indicates at least a mild to moderate delay.

#### 2.3.2 Construction of developmental patterns

In the second step of the analysis, we constructed developmental patterns for cognitive, language, and motor skills based on the average composite scores in each domain. We used the term “developmental patterns” to describe developmental changes over time, as this wording more accurately captures the descriptive nature of our analysis and better aligns with the methodological approach used in the study. Only participants who were assessed at both key time points—7 or 8 months corrected age and 24 or 25 months chronological age—were included in the pattern analysis. The average composite scores were interpreted using established Bayley-III cut-off values for developmental delay (Vohr et al., 2012). The level of delay at each time point was used to determine the direction of the developmental pattern in each domain. In the following step, we applied a similar approach to construct individual developmental patterns for each of the 17 participants, separately for the cognitive, language, and motor domains. This analysis revealed three distinct pattern types for cognitive skills and four for both language and motor skills. For clarity, we calculated the average composite scores at both time points within each identified pattern type and created figures illustrating these patterns. We additionally calculated the proportion of participants in each pattern type and visualized all individual developmental paths across the three domains. This procedure was chosen for several reasons. First, it reflects our research design, which is longitudinal and descriptive in nature. Second, it is based on the understanding that early childhood development is not a linear process. We aimed to illustrate this by displaying the developmental paths of individual ELGA children using composite score data not only at the initial and final assessment points, but also at other intermediate measurement points throughout the second year of life. This approach allowed us to capture the evolution of developmental skills over time in more detail. Within the descriptive framework, our objective was to illustrate the dynamic nature of development between 7 months of corrected age and 25 months of chronological age for each ELGA child. To gain deeper insight into the patterns of individual developmental patterns within our ELGA sample, we examined their associations with key perinatal characteristics, specifically gestational age and birth weight.

#### 2.3.3 Bayley-III administration and clinical considerations

Finally, we would like to note that during the assessment conducted at 24 or 25 months of chronological age, age correction for prematurity was not applied when using the Bayley-III. This decision was based on two main factors: first, we followed the Bayley-III guidelines, which recommend applying age correction only up to 24 months of age (Bayley, 2006). Second, we aimed to achieve a more realistic assessment of developmental outcomes. This approach was informed by previous criticisms of the Bayley-III, which has been shown to underestimate developmental delay and overestimate abilities in infants with a birth weight under 1,000 grams at 2 years of age (Anderson et al., 2010; Vohr et al., 2012). Despite these concerns, the Bayley-III remains the most commonly used assessment tool in neonatal intensive care unit (NICU) follow-up programs, and its results continue to serve as a basis for referrals to early intervention services (Greene et al., 2013). The administration of the Bayley-III assessments was conducted by certified professionals (Oľga Matušková and Erika Jurišová), as expert administration and accurate clinical judgment of task performance are essential.

## 3 Results

### 3.1 Developmental functioning of cognition, language and motor skills

To address the primary objective of this study, which was to evaluate the development of cognitive, language, and motor skills, as well as the prevalence of developmental delays in Slovak ELGA children up to 2 years of age, we analyzed developmental outcomes in our sample (*n* = 17) at two time points based on composite scores: at 7 or 8 months corrected age and at 24 or 25 months chronological age.

As shown by the descriptive data presented in Table 1, the mean composite scores indicate that at 7 or 8 months of corrected age, the overall developmental functioning of ELGA children fell within the average range. However, ELGA children scored 5 to 10 points lower than the normative population across all domains, with motor skills demonstrating the greatest developmental vulnerability.

**Table 1:** Descriptive analyses of Bayley-III composite scores (*n* = 17).

	7th or 8th month of corrected age			24th or 25th month of age		
	M ± SD	Mdn	95% IC	M ± SD	Mdn	95% IC
Cognitive skills	94.1 ± 11.2	95.0	88.3–99.8	88.8 ± 13.9	90.0	81.6–96.0
Language skills	91.6 ± 9.5	94.0	86.5–96.5	83.5 ± 11.8	86.0	77.4–89.6
Motor skills	90.8 ± 11.4	91.0	84.9–96.7	87.5 ± 13.8	91.0	80.4–94.7

At 7 or 8 months of corrected age, the distribution of developmental functioning among ELGA children according to Bayley-III categories was as follows: no delay—cognition: 88.2% (*n* = 15), language: 88.2% (*n* = 15), motor: 70.5% (*n* = 12); mild to moderate delay (< −1 SD)—cognition: 11.7% (*n* = 2), language: 11.7% (*n* = 2), motor: 29.4% (*n* = 5). No cases of severe delay (< −2 SD) were observed in our sample. Seventy point 5 % (*n* = 12) of the ELGA children demonstrated no developmental delay across all three domains at 7 or 8 months corrected age. Delays in one domain were observed in 17.6% (*n* = 3) of the children, delays in two domains in 5.8% (*n* = 1), and delays across all three domains were present in 5.8% (*n* = 1) of the sample.

At 24 or 25 months of chronological age, the overall developmental functioning of ELGA children was within the low average range. ELGA children scored 12 to 17 points lower than the normative population across all domains, with language skills showing the greatest developmental vulnerability. ELGA children in their 24th or 25th month of age achieved the following distribution in developmental functioning according to Bayley-III scales: No delay: cognition: 58.8% (*n* = 10), language: 52.9% (*n* = 9), motor: 64.7% (*n* = 11). Mild to moderate delay (< −1 SD): cognition: 29.4% (*n* = 5), language: 29.4% (*n* = 5), motor: 17.6% (*n* = 3). Severe delay (< −2 SD): cognition: 11.7% (*n* = 2), language: 17.6% (*n* = 3), motor: 17.6% (*n* = 3). In the sample of ELGA children, we found overall developmental delay (−1 SD and −2 SD) most frequently in achieving language developmental milestones (47.0%, *n* = 8), in cognition (41.1%, *n* = 7), and least in motor skills (35.2%, *n* = 6).

Developmental functioning without delay in all three domains in the 2nd year was achieved by 41.1% (*n* = 7) of the ELGA children in the sample. At 2 years of age, delays were observed in 17.6% (*n* = 3) of ELGA children in one developmental domain, while another 17.6% (*n* = 3) had delays in two domains. Delays across all three domains were present in 23.5% (*n* = 4) of the children.

**Figure 1:**
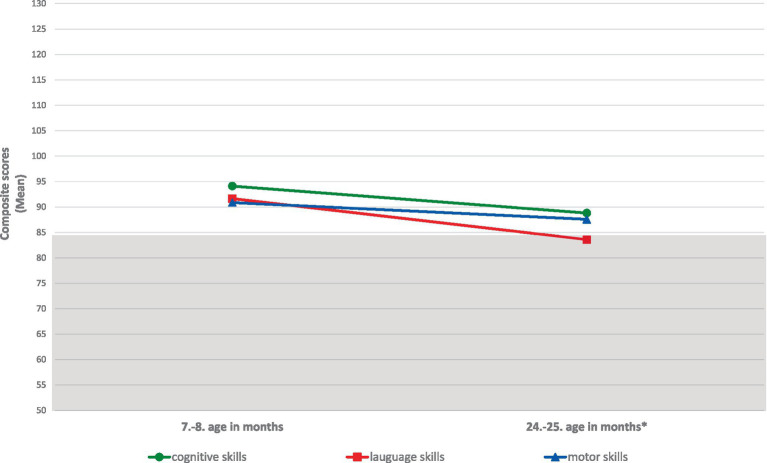
Developmental functioning of cognition, language and motor skills in ELGA children up to 2 years of age. *Composite scores at the final assessment were calculated without age correction for prematurity. **A composite score below 85 indicates developmental delay according to Bayley-III criteria.

Figure 1 illustrates the development of cognitive, language, and motor skills at two time points based on the average composite scores—at 7 or 8 months of corrected age and at 24 or 25 months of chronological age—in our sample of ELGA children. A decline in developmental functioning is observed in the cognitive and motor domains; however, the scores remain within the average range, without delay. In contrast, language skills show the most pronounced decline over the course of development up to 2 years, shifting from the average range to the low average range, which corresponds to mild to moderate developmental delay (< −1 SD).

### 3.2 Types of developmental patterns of cognitive, language and motor skills

The second aim of the study was to characterize the different types of developmental patterns in cognitive, language, and motor skills among 17 children with ELGA, assessed from 7 or 8 months of corrected age up to 24 or 25 months of chronological age.

We identified four distinct developmental pattern types across cognitive, language, and motor domains in these children: (1) Ascending to the normal range (resilient, demonstrating a “catch-up” effect); (2) Stable within the normal range; (3) Stable within the delayed range; and (4) Descending to the delayed range. The names of the patterns reflect the level of developmental delay observed at the initial and final assessment points. Patterns 1 and 2 were classified as developmentally favorable, indicating typical or improving development. In contrast, patterns 3 and 4 were considered developmentally at risk, reflecting persistent or worsening delays.

The percentage distribution of developmental patterns across the described types for cognition, language, and motor skills was as follows:

(1) ascending pattern: 0% (*n* = 0), 5.8% (*n* = 1), and 11.7% (*n* = 2);
(2) stable pattern within the normal range: 58.8% (*n* = 10), 47.0% (*n* = 8), and 52.9% (*n* = 9);
(3) stable pattern within the delayed range: 11.7% (*n* = 2), 5.8% (*n* = 1), and 17.6% (*n* = 3);
(4) descending pattern: 29.4% (*n* = 5), 41.1% (*n* = 7), and 17.6% (*n* = 3).

**Figure 2:**
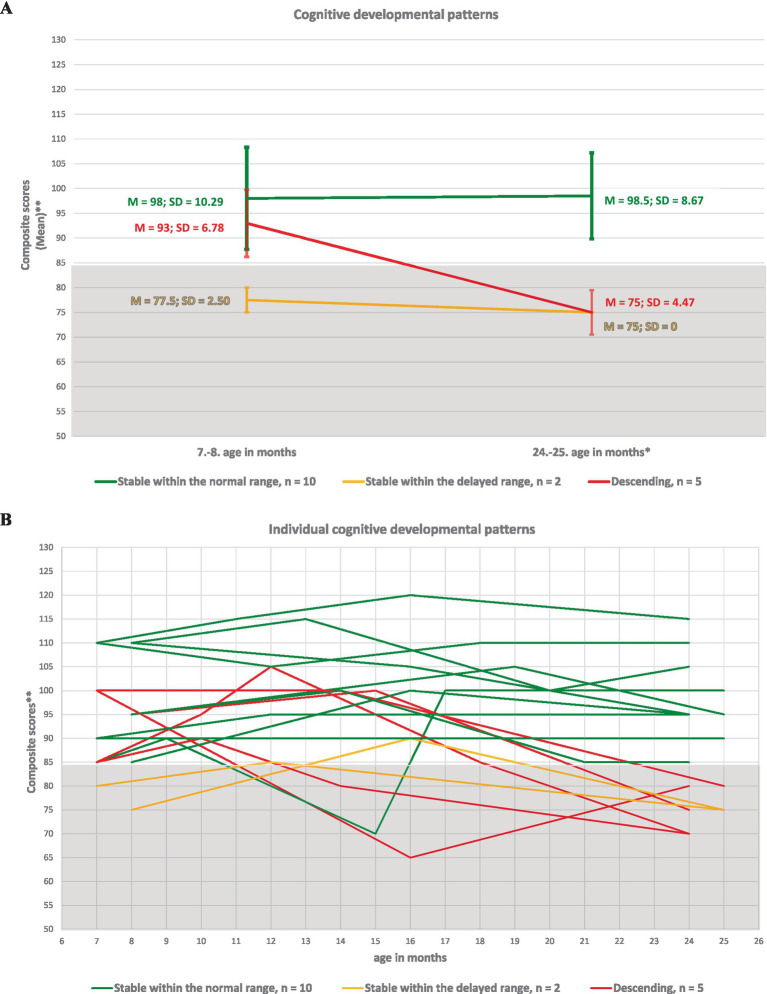
Developmental patterns of cognitive skills in ELGA children. **(A)** Group-level cognitive patterns based on the average composite scores at the first and last assessment points. **(B)** Individual cognitive patterns for all 17 participants. *Composite scores are presented without age correction for prematurity. **A composite score below 85 indicates developmental delay according to Bayley-III criteria.

**Figure 3:**
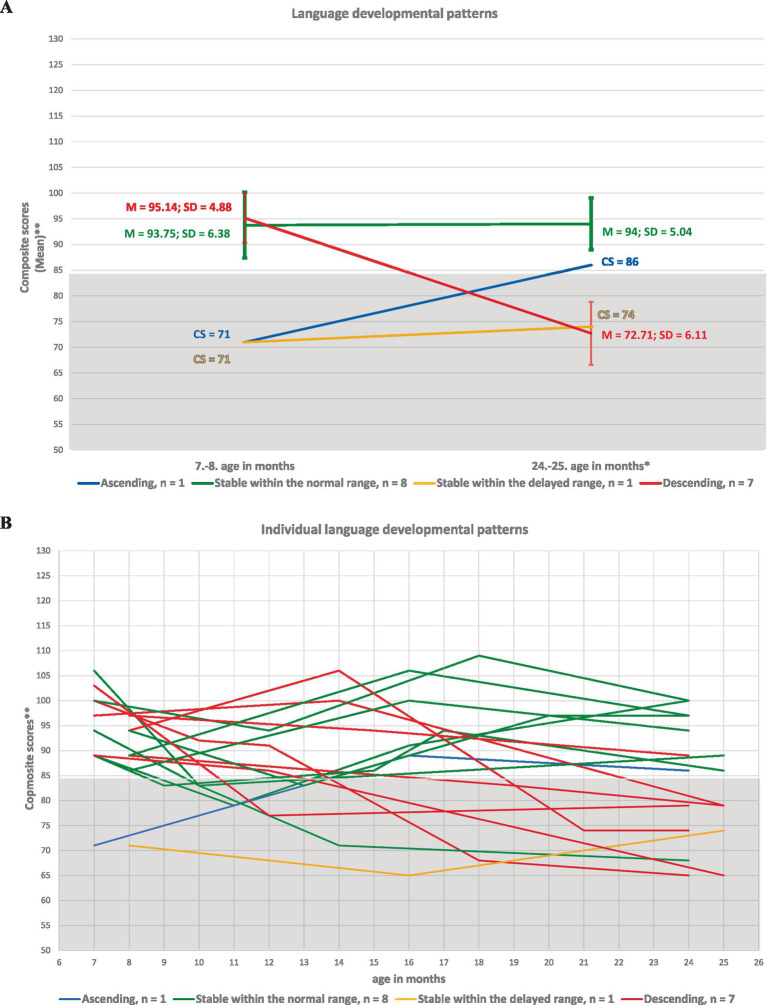
Developmental patterns of language skills in ELGA children. **(A)** Group-level language patterns based on the average composite scores at the first and last assessment points. **(B)** Individual language patterns for all 17 participants. *Composite scores are presented without age correction for prematurity. ** A composite score (CS) below 85 indicates developmental delay according to Bayley-III criteria.

**Figure 4:**
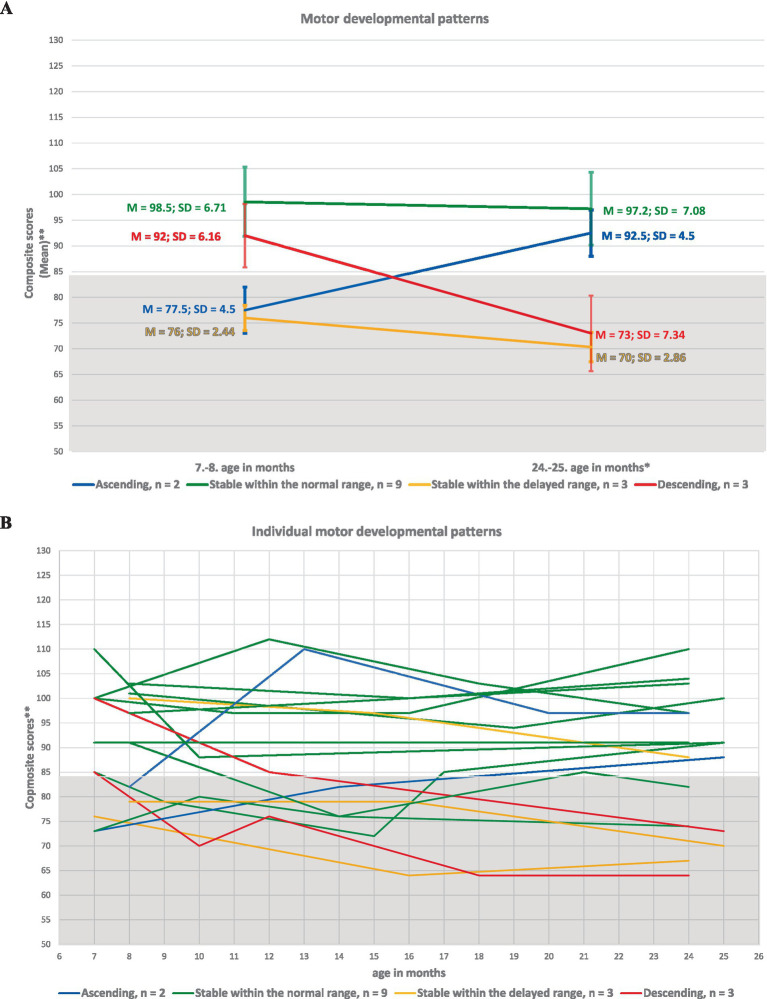
Developmental patterns of motor skills in ELGA children. **(A)** Group-level motor patterns based on the average composite scores at the first and last assessment points. **(B)** Individual motor patterns for all 17 participants. *Composite scores are presented without age correction for prematurity. **A composite score below 85 indicates developmental delay according to Bayley-III criteria.

The identified of developmental patterns for cognitive, language, and motor skills in ELGA children are illustrated in Figures 2–4.

### 3.3 Cognitive, language and motor developmental patterns from the perspective of gestational age and birth weight

In the next step, we took a closer look at the cognitive, language, and motor patterns of the observed ELGA children from the perspective of their gestation age and birth weight. The sample was stratified based on gestational age into distinct groups: preterm birth in 24.–25. GA: *n* = 7, and 26.–28. GA: *n* = 10. The stratification of the sample according to birth weight was as follows: < 1,500 g VLBW: *n* = 5; < 1,000 g ELBW: *n* = 6; < 750 g VELBW: *n* = 5; and < 600 g: *n* = 1.

#### 3.3.1 Cognitive developmental patterns

The percentage of ELGA children with different gestational age and birth weight in various developmental patterns of cognitive skills is presented in Table 2. Among ELGA children born at 24–25 weeks of GA the most represented patterns were: descending pattern, and the stable pattern within the delayed range. One child from this GA category had a stable pattern of cognitive skills within the normal range, being a child born at 25 weeks of GA, but with a higher BW of 740 g. Among ELGA children born at 26–28 weeks of GA the most represented was the stable pattern within the normal range. For one child from this GA category, a descending pattern was described. This child was born at 27 weeks of GA, but his BW was lower, 560 g. Among children with higher BW—VLBW and ELBW—we frequently described developmentally favorable trends (stable pattern within the normal range), while for children with BW < 750 g, the patterns were considered developmentally risky.

**Table 2:** Representation of participants with different GA and BW in various developmental patterns of cognitive skills.

		Developmentally favorable patterns		Developmentally risky patterns	
		Ascending	Stable within the normal range	Stable within the delayed range	Descending
GA	26. – 28. GA (*n* = 10)	0 (0%)	9 (90.0%)	0 (0%)	1 (10%)
	24. – 25. GA (*n* = 7)	0 (0%)	1 (14.2%)	2 (28.5%)	4 (57.1%)
BW	<1,500 g VLBW (*n* = 5)	0 (0%)	4 (80.0%)	0 (0%)	1 (20.0%)
	<1,000 g ELBW (*n* = 6)	0 (0%)	4 (66.6%)	0 (0%)	2 (33.3%)
	<750 g VELBW (*n* = 5)	0 (0%)	2 (40.0%)	2 (40.0%)	1 (20.0%)
	< 600 g (*n* = 1)	0 (0%)	0 (0%)	0 (0%)	1 (100%)

GA, gestational age; BW, birth weight; VLBW, Very low birth weight infants; ELBW, Extremely low birth weight infants; VELBW, Very extremely low birth weight infants; < 600 g, fetal infants.

#### 3.3.2 Language developmental patterns

The percentage of the observed ELGA children with different GA and BW in the various developmental patterns of language skills is presented in Table 3. Among ELGA children born at 24–25 weeks of GA the most represented patterns were: considered developmentally risky, particularly the descending pattern, and stable pattern within the delayed range. From this GA category, two children demonstrated developmentally favorable patterns: one with a BW of 860 g (born at 25 weeks of GA, demonstrating a „catch up“pattern) and another with a BW of 690 g (born at 24 weeks of GA, stable within the normal range). Among ELGA children born at 26–28 weeks of GA the most represented pattern was the stable pattern within the normal range. For three children from this GA category, we described a descending pattern. These included children born with a BW of 560 g (27 weeks GA), as well as children with higher birth weights: 1,000 g (28 weeks GA) and 1,070 g (26 weeks GA). Among children with higher birth weights—VLBW and ELBW—we more frequently described developmentally favorable trends (stable pattern within the normal range), while for children with BW < 750 g, the patterns were considered developmentally risky.

**Table 3:** Representation of participants with different GA and BW in the various developmental patterns of language skills.

		Developmentally favorable patterns		Developmentally risky patterns	
		Ascending	Stable within the normal range	Stable within the delayed range	Descending
GA	26. – 28. GA (*n* = 10)	0 (0%)	7 (70.0%)	0 (0%)	3 (30.0%)
	24. – 25. GA (*n* = 7)	1 (14.2%)	1 (14.2%)	1 (14.2%)	4 (57.1%)
BW	<1,500 g VLBW (*n* = 5)	0 (0%)	3 (60.0%)	0 (0%)	2 (40.0%)
	<1,000 g ELBW (*n* = 6)	1 (16.6%)	3 (50.0%)	0 (0%)	2 (33.3%)
	<750 g VELBW (*n* = 5)	0 (0%)	2 (40.0%)	1 (20.0%)	2 (40.0%)
	< 600 g (*n* = 1)	0 (0%)	0 (0%)	0 (0%)	1 (100%)

GA, gestational age; BW, birth weight; VLBW, Very low birth weight infants; ELBW, Extremely low birth weight infants; VELBW, Very extremely low birth weight infants; <600 g, fetal infants.

#### 3.3.3 Motor developmental patterns

The percentage of the observed ELGA children with different GA and BW in the various developmental patterns of motor skills is presented in Table 4.

**Table 4:** Representation of participants with different GA and BW in the various developmental patterns of motor skills.

		Developmentally favorable patterns		Developmentally risky patterns	
		Ascending	Stable within the normal range	Stable within the delayed range	Descending
GA	26. – 28. GA (*n* = 10)	1 (10.0%)	7 (70.0%)	1 (10.0%)	1 (10.0%)
	24. – 25. GA (*n* = 7)	1 (14.2%)	2 (28.5%)	2 (28.5%)	2 (28.5%)
BW	<1,500 g VLBW (*n* = 5)	1 (20.0%)	4 (80.0%)	0 (0%)	0 (0%)
	<1,000 g ELBW (*n* = 6)	1 (16.6%)	2 (33.3%)	1 (16.6%)	2 (33.3%)
	<750 g VELBW (*n* = 5)	0 (0%)	3 (60.0%)	1 (20.0%)	1 (20.0%)
	< 600 g (*n* = 1)	0 (0%)	0 (0%)	1 (100%)	0 (0%)

GA, gestational age; BW, birth weight; VLBW, Very low birth weight infants; ELBW, Extremely low birth weight infants; VELBW, Very extremely low birth weight infants; <600 g, fetal infants.

Among ELGA children born at 26–28 weeks of GA the most represented pattern was the stable pattern within the normal range. We observed a „catch-up“pattern in this category for one child (GA: 26 weeks, BW: 980 g). Despite the fact that among ELGA children born at 24–25 weeks of GA the percentage of considered developmentally risky patterns was higher: descending pattern and stable pattern within the delayed range no psychologically significant difference in comparison with the representation of developmentally favorable trends (as was the case with cognition and language) was identified. Among children with VLBW and VELBW, developmentally favorable trends were more frequently described. Among children with ELBW, the percentage of ELGA children with developmentally favorable and developmentally risky patterns was balanced. For one child with a BW less than 600 g, we found a developmentally risky pattern—stable within the delayed range.

## 4 Discussion

A shorter duration of physiological maturation in the fetus carries a high risk of adverse developmental outcomes (e.g., Johnson et al., 2011; Marlow et al., 2005; O'Shea et al., 2013; Sansavini et al., 2011; Sansavini et al., 2014; Peralta-Carcelen et al., 2017; Squarza et al., 2017) necessitating thorough examination to ensure early and appropriate stimulation for the child.

This study aimed to longitudinally examine neurodevelopmental patterns, the prevalence of developmental delays, and the risk factors (gestational age and birth weight) associated with cognitive, motor, and language development in 17 Slovak children born at extremely low gestational age, without major cerebral damage or severe visual impairments, up to 2 years of age. A descriptive approach was employed.

In the discussion, we approach the comparison of our findings with other studies cautiously, because individual studies of the developmental functioning of ELGA children can differ in many aspects, such as the research methodology, research methods, sample size, age of children at the time of testing, use of age correction for prematurity, presence of a control group, and the degree of strictness of exclusion criteria for selecting participants, such as the presence of major cerebral damage or severe visual impairments. Being cautious in drawing conclusions based on our findings is essential mainly because of the study design, which was longitudinal, but due to the small number of participants, a descriptive approach was used.

### 4.1 Developmental functioning of cognition, language and motor skills of ELGA children up to 2 years of age

The developmental functioning of cognitive, language, and motor skills in the sample of ELGA children studied at the 7th or 8th month of corrected age fell within the average range. At this stage, the greatest developmental vulnerability was identified in the domain of motor skills. In the ELGA children studied, we observed a decline in cognitive, language, and motor functioning to the low-average range at 2 years of chronological age. Among ELGA children, language scores were the lowest. Sansavini et al. (2014) also observed similar mean composite Bayley-III scores across individual domains at 24 months of corrected age. Were comparable to our findings at 24 months of uncorrected age, with the following values (mean, SD): cognition: 85.3 (7.4) [Sansavini et al., 2014]—88.8 (13.9) [our study]; language: 93.8 (12.9)—83.5 (11.8); and motor: 85.2 (6.7)—87.5 (13.8). In both studies, the number of participants was the same (*n* = 17), and the subjects were ELGA children without major cerebral damage or severe visual impairments. This finding raises the question of whether age correction for prematurity remains necessary at 24 months and beyond, particularly when conducting developmental assessments using the Bayley-III in preterm children without major cerebral damage or severe visual impairments. We examine this question in consideration of concerns about the potential overestimation of performance when using the Bayley-III.

At 2 years of chronological age, the prevalence of developmental delay in individual domains among ELGA children in our sample was as follows: Mild to moderate delay (< −1 SD): cognition: 29.4%; language: 29.4%; motor: 17.6%. Severe delay (< −2 SD): cognition: 11.7%; language: 17.6%; motor: 17.6%. Comparable results were reported in the Swedish Preterm Infants Study (EXPRESS), which found that the preterm group performed significantly lower than the control group on the Bayley-III subtests at 2.5 years of corrected age. The study also included ELGA children with major neonatal morbidities and sensorimotor impairment. The prevalence of moderate–severe delay (< −2 SD) was 10.8% in cognitive domain, 14.9% in receptive communication, 14.5% in expressive communication, 12.4% in fine motor, and 7.0% in gross motor functions (Månsson and Stjernqvist, 2014).

When examining the development of ELGA children across the observed domains from the 7th or 8th month of corrected age to the 24th or 25th month of chronological age, a decline in developmental functioning is evident across all domains. This decline is reflected in several indicators: (1) a decrease in the average composite score over time, (2) an increasing gap in average scores between our sample and the normative population, (3) a rise in the prevalence of developmental delays, and (4) a deepening severity of developmental delays over time. In the sample of ELGA children, we identified a declining developmental trends in cognitive and motor skills; however, it remained within the range of typical development without delay. Regarding language skills, the most significant decline was observed over the course of development up to 2 years of age, shifting from the range of typical development to mild to moderate delay (< −1 SD).

It is important to emphasize that as early as the 7th and 8th month of corrected age, despite their average composite scores falling within the typical range across all developmental domains, ELGA children scored 5 to 10 points lower than the normative population across domains. This gap continued to widen over time, and by 2 years of chronological age, ELGA children scored 12 to 17 points lower than the normative population across domains. In addition to the increasing gap in average scores compared to the normative population, the growing difference in skill levels over time is also reflected in the rising prevalence and severity of developmental delay among ELGA children. Longitudinally, we found that while at the 7th or 8th month of corrected age, delays were exclusively at the level of mild to moderate delay (< −1 SD), by 2 years of chronological age, delays were identified not only at the mild to moderate level (< −1 SD) but also at the severe level (< −2 SD) across all domains. We also found that while only 30% of ELGA children exhibited developmental delay in at least one domain at the 7th or 8th month of corrected age, this percentage had risen to 58.9% by 2 years of chronological age. Our findings align with data from Hutchinson et al. (2013), who reported a 70% rate of impairment in one or more neurodevelopmental domains among ELGA children. Our results indicate a growing divergence in development, particularly in language skills, among the studied group of ELGA children up to 2 years of age. The findings of this study support the hypothesis of the so-called Matthew effect, observed in the developmental trajectories of preterm children compared to term-born children, as highlighted by Sansavini et al. (2014).

Our results also suggest the presence of the so-called cascading effect between developmental functions in the studied sample of ELGA children. A total of 23.5% of ELGA children in their second year of life exhibited delays across all developmental domains (cognitive, language, and motor). This finding suggests that more than half of the ELGA children studied in their second year exhibited some degree of developmental delay. Sansavini et al. (2010) highlight that extremely low and very low gestational age can serve as a risk factor for development, even in the absence of cerebral damage, as an immature central nervous system is exposed to invasive and inadequate stimulation. Preterm birth can result in subtle cerebral neuropathologies (Volpe, 1981; Rees and Inder, 2005) and subsequent long-term physical and neurological complications (Buonocore et al., 2001). Our findings also support the hypothesis that the development of individual functions is intertwined and interrelated.

In the ELGA children studied, we observed a decline in cognitive, language, and motor functioning to the low-average range at 2 years of chronological age. The language scores were the lowest, falling into the range of mild to moderate delay (< −1 SD). It appears that some of the difficulties identified in the language skills of preterm children may be mediated by general cognitive functions (Ortiz-Mantilla et al., 2008; Rose et al., 2009; Van Lierde et al., 2009), but they may also be related to some aspects of motor and visual development (Sansavini et al., 2011). Selassie et al. (2005) report that premature infants often have impaired gross and fine motor development, including oromotor development, which affects the movements of the speech organs and leads to difficulties not only in speech development but also in feeding. It can also be related to the cascading effect between functions, where the insufficient development of a certain function (cognitive, language, motor) negatively affects the achievement of developmental milestones in other functions. Early stimulation of motor skills in ELGA children can bring benefits not only for motor skills but also for cognitive and linguistic skills grounded on motor skills (Sansavini et al., 2014). Understanding the extent of developmental delays and their covariation across developmental domains is crucial for improving and optimizing clinical practice. These findings underscore the importance of providing early multidisciplinary care for ELGA children, particularly from their second year of life, with a focus on recognizing developmental interdependencies, such as the relationship between language and other developmental areas.

### 4.2 Types of developmental patterns of cognitive, language and motor skills of ELGA children up to 2 years of age

The study also aimed to enhance understanding of the diverse developmental patterns shaping the early development of cognition, language, and motor skills in ELGA children during their first 2 years of life. We described four types of developmental patterns: (1) stable within the normal range; (2) ascending into the normal range (resilient, with a catch-up effect); (3) stable within the delayed range; and (4) descending into the delayed range.

#### 4.2.1 Stable developmental patterns

The most commonly observed pattern across all domains was the stable pattern within the normal range (cognitive—58.8%, language—47.0%, and motor—52.9%). We assume that this result may be attributed to the fact that the children in our sample had no severe cerebral damage or severe visual impairments. Furthermore, despite perinatal risks, they developed well from the beginning, having their developmental scores within the normal range and without delay.

This may also explain the finding of the low representation of the stable patterns within the delayed across all developmental domains in our sample (cognitive—11.7%, language—5.8%, and motor—17.6%). Several authors have also identified stable developmental patterns, and trajectories of functioning in various domains in preterm children. Stable developmental trajectories were identified within the normal range, within the deficit range, and also stable trajectories within the normal range but with continuous lagging compared to the control group of full-term children.

Stålnacke et al. (2015) conducted a cluster analysis of cognitive skills in a sample of 118 preterm children (< 37 weeks of GA and < 1,500 g) at the time points of 10 months, 5.5 years, and 18 years. Most children remained within clusters representing similar developmental levels over time, with transitions between lower and higher clusters occurring only rarely. Children who were classified in the lower “cognitive class” at 5.5 years of age did not demonstrate catch-up effect in their cognitive development. According to the study’s conclusions, it can be assumed that the cognitive trajectories of preterm children have a stable nature; and cognitive performance at 5.5 years was a strong predictor of cognitive performance at 18 years.

Mangin et al. (2017) evaluated white matter anomalies in 110 very preterm infants (≤ 32 weeks of GA) and a group of full-term children using magnetic resonance imaging (MRI), in addition to psychological testing of cognitive skills. Testing was conducted four times (at 4, 6, 9, and 12 years). Cognitive skill trajectories in both groups showed stability over time, but the preterm children scored 9–12 points lower than the full-term children in each test. Lower IQ scores in both groups were associated with higher levels of social risk, and in preterm children, also with a higher degree of white matter anomalies.

We consider the study by Sansavini et al. (2014) methodologically stimulating. They compared the growth curves of cognition, language, and motor skills in 17 ELGA children (mean GA 25.7 weeks) and 11 full-term children at various time points from 12 to 36 months of GA. Growth curves were used to model the raw scores in the individual domains of the Bayley-III. Children born at ELGA scored significantly lower than their full-term peers in language, motor, and cognitive skills. By the age of 3 years, they had not overcome this disadvantage, even when their corrected age was considered. Regarding growth trajectories, a significant and increasing divergence was observed in motor development, with the ELGA group increasingly lagging behind their full-term peers counterparts over time. In linguistic and cognitive development, a stable gap between the two samples was observed. Additionally, substantial interindividual variability in the rate of language development was observed in both groups.

In the motor domain, our sample exhibited the highest representation of a stable pattern within the delayed range, compared to the cognitive and language domains. As previously noted, the motor domain appears particularly vulnerable to the effects of preterm birth. Drawing on the neuroconstructivist framework (Karmiloff-Smith, 1998; Thomas et al., 2009), we propose that the motor deficits observed in this study may result from the interaction between the extreme immaturity of the nervous system and other body systems, combined with the nature of physical experiences during prolonged recovery in the NICU and the post-discharge period (Als et al., 2004; Sansavini et al., 2010; Sansavini et al., 2011).

#### 4.2.2 Descending patterns into the delayed range

The second most prevalent developmental patterns identified in the language domain (41.1%) and cognitive skills (29.4%) exhibited a descending pattern, characterized by a shift from typical to delayed performance. This pattern reflects a phenomenon in which preterm children’s developmental progress declines over time. A possible underlying mechanism may involve the interaction between the child’s smaller physical size and the initially lower complexity of tasks, reduced demands for cooperation, and less mature psychological processes required for performance in language, cognitive, and motor domains. As the child matures, this early immaturity increasingly impedes the development of more complex skills. We propose that assessments conducted at later ages are more likely to reveal developmental challenges, as task demands become more sophisticated. Across developmental stages, these deficits tend to intensify, resulting in a widening gap in performance between preterm and full-term children.

Nguyen et al. (2018) described developmental trajectories of language skills in 224 preterm children (< 30 weeks of GA or < 1,250 g) aged 2 to 13 years. In addition to the optimal/stable pattern, they identified three lagging trajectories: “decelerating” (in 9% of children)—with a good initial level of language skills, but gradual weakening from the age of 7 (the authors suggest that this could be due to overestimation of early language development by parents), “stable low” (21% of children), and “high risk” (7% of children). The authors suggest that if language skills have not improved by the age of seven, difficulties are likely to persist into adulthood. Preterm children were 8 times more likely to have poorer language development than full-term children.

Sansavini et al. (2010) examined the rate of language impairment among very preterm children, finding that about one-third of these children could be characterized as having a language impairment at 3.5 years. The predominant predictor of language impairment was a prior history of communicative and linguistic skills as reported at 2.5 years. The authors suggest that these findings point to the existence of specific subgroups of preterm children who are particularly vulnerable to persistent developmental difficulties. In our sample, 41.1% of ELGA children showed a descending pattern in language development by the age of two, while 47% exhibited a language development delay at that age. Based on the conclusions of Sansavini et al. (2010), it is possible to consider these subgroups as specific from the perspective of developmental risk for the language development of ELGA children.

Studies have confirmed significant increasing divergence in language skills over time, such as in Sansavini et al. (2011), which examined a sample of 104 Italian very preterm children (mean 29.5 weeks of GA). The effect was evident in receptive vocabulary and gesture/action production from 12 to 18 months and in expressive vocabulary from 18 to 24 months. A meta-analysis (van der Noort-Spek et al., 2012) revealed that very preterm children at preschool and school age show deficits in both: simple and complex language functions. In adolescence, they show a catch-up effect in some simple language functions, but they continue to have difficulties in complex ones (Luu et al., 2011).

Regarding the observed descending pattern of cognitive skills in our sample of ELGA children, we associate this finding with the hypothesis proposed by Rose et al. (2009, 2011). They propose that fundamental information-processing mechanisms such as processing speed and memory are impaired in preterm infants due to differences in neurobiological maturation. These impairments, in turn, may negatively impact the development of more complex cognitive functions. Findings from previous studies also indicate a worsening cognitive development in preterm children. For instance, Yaari et al. (2018) found that differences between the developmental trajectories of ELGA children and full-term peers continued to increase up to 18 months of corrected age, with the most pronounced differences observed in visual reception, gross motor, and fine motor skills. Meta-analyses have demonstrated that preterm children exhibit developmental differences in general intellect compared to full-term peers, with a difference of approximately 10 standard IQ points (Bhutta et al., 2002). This cognitive difference was more pronounced in ELGA children compared to those with lower degrees of prematurity (Marlow et al., 2005; Larroque et al., 2008) and was observed even when individuals with severe neurological impairments were excluded from the preterm sample (Charkaluk et al., 2010). In a longitudinal study, Sansavini et al. (2014) found that ELGA children exhibited significantly lower scores in language, motor, and cognitive skills compared to their full-term peers. Moreover, they did not overcome this disadvantage by the age of three, even when their corrected age was considered. Regarding growth curves, motor development showed a significant increasing divergence, demonstrating a Matthew effect, with the preterm sample falling further behind the full-term sample. In linguistic and cognitive development, however, a stable gap between the two samples was observed. Sansavini et al. (2010) report that cognitive difficulties in ELGA children become more apparent and stable later in development and persist throughout preschool years and subsequently into school age, with an increase in learning difficulties and disorders (Constable et al., 2008). Given these findings from longitudinal research, it is essential to provide appropriate intervention for ELGA children not only those already exhibiting delays (< −1 SD; < −2 SD), but also those showing a decline in cognitive skills compared to their full-term peers. Our clinical practice confirms this necessity, as these developmentally high-risk children face the potential persistence and deepening of cognitive differences.

#### 4.2.3 The ascending patterns

The ascending pattern was marginally represented in our sample of ELGA children (cognitive—0%; language—5.8%, and motor—11.7%). The catch-up effect in developmental functioning across various domains in preterm children has been confirmed by several studies. Luu et al. (2009) found a catch-up effect in receptive communication in children with very low birth weight (600–1,250 g). Similarly, Nguyen et al. (2018) described five different language skill trajectories in 224 very preterm children (< 30 weeks of GA or < 1,250 g) aged 2–13 years. The most prevalent developmental pattern, designated by the authors as optimal/stable, comprised two distinct patterns: a stable within the normal range pattern, observed in 23% of children, and a resilient pattern characterized by an initial delay followed by a catch-up effect identified in 37% of children. The remaining trajectories were categorized as decelerating, stable low, and high risk. In the study by Everts et al. (2019), the group of premature children even outperformed the full-term children in the areas of inhibition and cognitive flexibility (the testing was conducted at two time points, between the ages of 7–12 and 13–18). These findings support the hypothesis that preterm children require an extended period for the maturation of executive functions, with this developmental process continuing into early adulthood.

The study by Syrengelas et al. (2016), focusing on the motor development of preterm children, revealed an upward developmental trend. The children were assessed monthly using the Alberta Infant Motor Scale (AIMS) from birth until 19 months of GA. In the prone position (face-down on their belly), preterm children had significantly lower gross motor scores compared to full-term children up to 12–13 months. In the supine position (lying on their back with their face pointing upward), differences were again observed in favor of full-term children up to 8–9 months, followed by a plateau phase during which preterm children caught up with their full-term peers. In the sitting position, differences were noted from 3 to 4 months, while the development of standing phases varied from the beginning. However, the motor development trajectories of preterm children closely resembled those of the control group. The authors concluded that while the motor development of preterm children follows a typical pattern, it progresses at a slower rate. In our ELGA sample, developmental functioning was assessed at the second time point at 2 years of age raising the possibility that a catch-up effect may emerge at a later stage.

### 4.3 Developmental patterns of cognitive, language and motor skills from the perspective of gestational age and birth weight of ELGA children up to 2 years of age

This study also aimed to gain deeper insights into the developmental patterns across cognitive, language, and motor domains within the ELGA sample. To this end, we examined their associations with key perinatal characteristics specifically gestational age and birth weight. This analysis was guided by previous research identifying these factors as strong predictors of neurodevelopmental outcomes in preterm populations (Salas et al., 2016; Su et al., 2017; UCSF Benioff Children’s Hospital, 2025). Our findings confirm that, despite advancements in perinatal care for premature infants, low gestational age and very low birth weight or lower continue to pose significant risks to early childhood development.

In our sample of ELGA children, those born at 24–25 weeks of gestation predominantly exhibited developmental patterns classified as high-risk, including descending patterns and stable patterns within the delayed developmental range. These patterns were particularly prevalent in cognitive and language domains. Conversely, among children born at higher gestational ages (26–28 weeks of GA), stable developmental patterns within the normal range predominated across all domains. Neurodevelopmental outcomes improved incrementally with each additional week of gestational age.

Regarding birth weight, children with higher birth weight—very low birth weight infants (< 1,500 g) and extremely low birth weight infants (< 1,000 g)—frequently exhibited developmentally favourable trends (stable pattern within the normal range), while for children with birth weight < 750 g, the patterns were considered developmentally risky. This trend was observed across all developmental domains. Using a descriptive approach and analysis of developmental patterns in ELGA children, we identified a potentially protective effect of birth weight on neurodevelopment, particularly in the domains of cognition and speech. For example, we identified descending developmental patterns in cognition and speech in a child born at 27 weeks of GA, but with a birth weight of 560 g. Conversely, in a child born at a lower GA (25 weeks of GA) but with a higher birth weight (740 g), we identified stable patterns within the normal range across all developmental domains.

There appears to be an inverse relationship persists between birth weight or gestational age and the incidence of neurodevelopmental disorders, with lower birth weights and earlier gestational ages associated with higher rates of impairment (Bhutta et al., 2002). Each additional week of gestational age or gram of birth weight has been associated with a decreased likelihood of impairment (Synnes et al., 1994) and improved functional outcomes (MacDonald, 2002), particularly in cognitive development (Fily et al., 2006; Piecuch et al., 1997).

While some researchers consider birth weight a reliable predictor of neonatal outcomes (Cole et al., 2002), and others emphasize the importance of gestational age (Cooke, 2005; Foster-Cohen et al., 2007; Saigal and Doyle, 2008), Tyson et al. (2008) argue that neither factor alone serves as a robust predictor of developmental functioning in premature children. Instead, the authors emphasize that developmental outcomes are ultimately shaped by a complex interplay of medical and non-medical risk factors. There are proven interventions to help reduce stress caused by prematurity. These interventions aim to replicate some aspects of the intrauterine environment and enhance contact between the infant and caregiver, such as removing the infant from the incubator for skin-to-skin contact (kangaroo care) (Closa Monasterolo et al., 1998) or implementing the Newborn Individualized Developmental Care and Assessment Program (NIDCAP) (Als et al., 2004). Specifically, sensory stimulation and skin-to-skin contact have been shown to positively affect neurodevelopmental outcomes by improving sensory integration, strengthening parent-infant bonding, and supporting autonomic regulation (La Rosa et al., 2024; Lilliesköld et al., 2025; Ndjomo et al., 2025; Kristoffersen et al., 2005).

### 4.4 Limitations

We believe that the primary contribution of this study despite its small sample size lies in its findings on developmental levels, developmental patterns, and the presence of developmental delays among Slovak ELGA children during their second year of life. To our knowledge, no comparable study has been published in Slovakia to date. Nevertheless, we acknowledge several limitations of the study.

The primary limitation was the small sample size, which resulted from the strict inclusion and exclusion criteria applied during participant selection. These criteria were deliberately designed to isolate the effects of extreme prematurity on child development, minimizing the confounding influence of major cerebral damage or severe visual impairments. While this approach provided a clearer perspective on the developmental impact of extreme prematurity, it also substantially reduced the research sample size. Consequently, the generalizability of our findings beyond the study population should be interpreted with caution. Replicating these findings in a larger sample represents an important direction for future research. Although this study had a longitudinal aim, the small sample size necessitated a descriptive approach to examine the developmental patterns of Slovak ELGA children. The use of detailed, individual-level developmental profiles allows for a nuanced understanding of the variation within this high-risk group, offering insights that group-level statistics may overlook. Despite the small sample, the findings have direct clinical relevance for early developmental monitoring and intervention planning for extremely preterm infants.

Secondly, the study lacks a control group of term-born children. Instead, we compared the performance of ELGA children in our sample with a normative group that includes not only term-born children but also other developmentally at-risk populations [e.g., Down Syndrome, cerebral palsy, pervasive developmental disorder, premature birth, specific language impairment, prenatal alcohol exposure, asphyxiation at birth, small for gestational age, and at risk for developmental delay (Bayley, 2006)]. We did not establish a control group of full-term children, as our data were obtained through a retrospective analysis of medical records from patients selected according to the study’s criteria. We acknowledge that the use of test norms instead of raw scores from a control group may potentially reduce the observed differences in developmental outcomes across the assessed domains. However, the findings of Aylward and Zhu (2019) support our results. Their study demonstrated that the inclusion of at-risk children in the normative sample led to minimal changes in mean scores on the Cognitive, Language, and Motor Scales, suggesting that such inclusion did not significantly inflate Bayley-III scores.

Lastly, we acknowledge the absence of Slovak normative values for the Bayley-III as a limitation. We acknowledge the valid concern regarding the use of U.S.-based Bayley-III norms in a Slovak sample. While we recognize that cultural and contextual differences may influence developmental performance, we believe the application of Bayley-III norms in our study is justified for several reasons. First, in the absence of national normative data, the Bayley-III remains one of the most comprehensive and psychometrically validated tools for early developmental assessment globally. Applying the standard norms from the Bayley-III manual allowed us to maintain methodological rigor and comparability with international research. Second, we conducted a previous study in Slovakia with a sample of 30 children, where we examined the intercorrelations between Bayley-III subscales (Ráczová et al., 2024). The results showed even higher intercorrelations than those reported in the original U.S. standardization sample, suggesting good internal consistency in our context. While we acknowledge that this does not replace full psychometric standardization, it provides preliminary evidence supporting the instrument’s applicability in Slovak settings.

We also recognize that the use of non-local norms could influence the classification of developmental delay, especially among vulnerable groups such as extremely low gestational age (ELGA) infants. This limitation is addressed in our manuscript, and we interpret delay classifications with the necessary caution. Finally, this study is part of a broader effort to encourage the adaptation and standardization of high-quality developmental assessment tools for the Slovak population. The lack of national norms remains a major challenge in our context, and our research aims to contribute toward filling this gap and promoting further validation initiatives.

## 5 Conclusion

Our findings indicate that among the observed ELGA children, specifically those without significant cerebral damage or severe visual impairments and who received perinatal care, 41.1% showed no developmental delay at 2 years of age, even when assessed without age correction for prematurity. The most frequently observed developmental pattern across all domains (cognitive, language, and motor) was a favorable, stable pattern within the normal range. It appears that brain plasticity, potentially serving as a protective mechanism—enhanced by a complex interplay of medical and non-medical risk factors—may underlie the favourable neurocognitive development observed in preterm children (DeMaster et al., 2019). However, based on our clinical experience, we recommend closely monitoring the developmental patterns of these children, as delays may emerge later when confronted with more complex developmental tasks.

Conversely, more than half (58.9%) of the observed ELGA children at 2 years of age exhibited developmental delays in at least one domain. We more frequently identified developmental delays that were classified as mild to moderate in severity (< −1 SD). The second most common pattern in the domains of language and cognitive skills was the descending pattern, indicating a shift from typical development to developmental delay. Overall, across all developmental domains, we observed a general decline in performance levels into the low average range. In our study, cognitive and language skills appeared to be more developmentally vulnerable than motor skills among children without significant cerebral damage. Despite notable advancements in perinatal care, preterm children continue to face a high risk of developmental deficits.

The developmental patterns observed in ELGA children in this study reveal substantial interindividual variability in development up to 2 years of age. This variability appears to be influenced by the complex interplay of multiple factors, including the degree of prematurity measured by gestational age at birth and birth weight. These findings underscore the critical need for long-term monitoring of developmental patterns in this high-risk population. Furthermore, they highlight the importance of identifying a broad spectrum of medical, biological, and environmental risk factors and their interactions, that contribute to developmental outcomes in ELGA children without significant cerebral damage or severe visual impairments.

## Data Availability

The original contributions presented in the study are included in the article/supplementary material, further inquiries can be directed to the corresponding author.
